# Variability in Health Behaviors Over 14 Years Predicts Later Cognitive Outcomes

**DOI:** 10.1093/geroni/igaf122.2387

**Published:** 2025-12-31

**Authors:** Elayna Seago, Benjamin Katz, Laura Sands

**Affiliations:** Virginia Tech, Blacksburg, Virginia, United States; Virginia Tech, Blacksburg, Virginia, United States; Virginia Tech, Blacksburg, Virginia, United States

## Abstract

Engaging in healthy behaviors is important for maintaining cognitive functioning throughout the lifespan. We assessed whether variability in physical exercise and hours of sleep over a fourteen year period are associated with subsequent cognitive performance among Health and Retirement Study (HRS) participants aged 50-69 in 2004. We calculated variability in exercise and sleep across 7 biennial assessments by dividing the standard deviation of each participant’s biennial scores by the mean of the participant’s exercise or sleep score over the 14 year interval. Linear Regression analyses of participants aged 50-59 (n = 2370) at baseline revealed that higher initial cognition and educational levels, and female gender were associated with higher cognitive scores 14 years later. Although baseline levels of exercise and mobility were not associated with cognitive scores 14 years later, older age, more chronic conditions at baseline, and higher variability in exercise scores over the 14 years were negatively associated with cognitive scores in 2018. Similar results were found for participants aged 60-69 (n = 2738). We replicated the analyses described above to assess the association between variability in hours of sleep and subsequent cognitive functioning and found very similar findings. Importantly, we also found that the greater variability in biennial assessments of sleep was negatively associated with cognitive functioning 14 years later for those aged 50-59 (n = 743) at baseline, and 60-69 (n = 889) at baseline. The findings reveal the importance of considering variability in health behaviors when assessing their impact on future cognitive functioning and the importance of maintaining health behaviors throughout adulthood.

